# DBSecSys: a database of *Burkholderia mallei* secretion systems

**DOI:** 10.1186/1471-2105-15-244

**Published:** 2014-07-16

**Authors:** Vesna Memišević, Kamal Kumar, Li Cheng, Nela Zavaljevski, David DeShazer, Anders Wallqvist, Jaques Reifman

**Affiliations:** Department of Defense Biotechnology High Performance Computing Software Applications Institute, Telemedicine and Advanced Technology Research Center, U.S. Army Medical Research and Materiel Command, Fort Detrick, MD 21702 USA; Bacteriology Division, U.S. Army Medical Research Institute of Infectious Diseases, Fort Detrick, MD 21702 USA

**Keywords:** Bacterial secretion system, Virulence factors, Pathogenic mechanisms of action, Host-pathogen interactions, *Burkholderia mallei*

## Abstract

**Background:**

Bacterial pathogenicity represents a major public health concern worldwide. Secretion systems are a key component of bacterial pathogenicity, as they provide the means for bacterial proteins to penetrate host-cell membranes and insert themselves directly into the host cells’ cytosol. *Burkholderia mallei* is a Gram-negative bacterium that uses multiple secretion systems during its host infection life cycle. To date, the identities of secretion system proteins for *B. mallei* are not well known, and their pathogenic mechanisms of action and host factors are largely uncharacterized.

**Description:**

We present the **D**atabase of ***B****urkholderia mallei***Sec**retion **Sys**tems (DBSecSys), a compilation of manually curated and computationally predicted bacterial secretion system proteins and their host factors. Currently, DBSecSys contains comprehensive experimentally and computationally derived information about *B. mallei* strain ATCC 23344. The database includes 143 *B. mallei* proteins associated with five secretion systems, their 1,635 human and murine interacting targets, and the corresponding 2,400 host-*B. mallei* interactions. The database also includes information about 10 pathogenic mechanisms of action for *B. mallei* secretion system proteins inferred from the available literature. Additionally, DBSecSys provides details about 42 virulence attenuation experiments for 27 *B. mallei* secretion system proteins. Users interact with DBSecSys through a Web interface that allows for data browsing, querying, visualizing, and downloading.

**Conclusions:**

DBSecSys provides a comprehensive, systematically organized resource of experimental and computational data associated with *B. mallei* secretion systems. It provides the unique ability to study secretion systems not only through characterization of their corresponding pathogen proteins, but also through characterization of their host-interacting partners.

The database is available at https://applications.bhsai.org/dbsecsys.

## Background

### Introduction

Pathogenic bacteria cause a variety of diseases and represent a major public health concern worldwide. Secretion systems are a key component of bacterial pathogenicity, as they facilitate pathogenic internalization, survival, and replication within host cells through controlled transport and translocation of pathogenic molecules, e.g., proteins or DNA, from the interior of the bacterial cell into the host cell’s cytosol [1, 2]. Bacterial species frequently contain more than one type of secretion system, and some secretion systems are often encoded in multiple gene clusters (also called pathogenicity islands) [3–7].

The Gram-negative bacterium *Burkholderia mallei*, the causative agent of glanders, uses a variety of specialized secretion systems to interfere with host defense mechanisms and promote pathogen colonization. Given its ability to infect hosts via aerosol exposure, considerable antibiotic resistance, and the lack of a vaccine, *B. mallei* represents an emerging public health threat [8]. While the research into *B. mallei* pathogenicity has recently expanded, the identities of all of its secretion systems’ proteins are not completely cataloged and their pathogenic mechanisms are largely unknown.

To date, several database systems provide information about Burkholderia species proteins. Some provide general *B. mallei* protein information [9, 10], such as sequence annotation, naming schemes, and functional and pathway associations, but such systems lack systematic organization of the pathogen proteins that are part of secretion systems. Other databases are more specific and encompass information about single [11–13] or multiple [14–16] bacterial secretion systems for many, but not all, Burkholderia species. While these databases provide a useful characterization of proteins based on the secretion system type, they lack information about pathogenic mechanisms of action associated with secretion systems and their host targets.

### Our contribution

As none of the available secretion system databases includes *B. mallei* secretion systems data, the goal of the **D**atabase of ***B****urkholderia mallei***Sec**retion **Sys**tems (DBSecSys) is to provide information about *B. mallei* (strain ATCC 23344) proteins associated with secretion systems and their host factors. Currently, DBSecSys contains experimentally and computationally derived information about *B. mallei* proteins associated with secretion systems. The information includes their secretion system types, involvement in virulence, associated mechanisms of action, host targets (interacting host proteins), and general annotation (assigned names and IDs, protein sequence, and functional and pathway association). These features provide researchers with the unique ability to study *B. mallei* pathogenicity and secretion systems not only through characterization of the corresponding pathogen proteins, but also through characterization of their host interacting partners.

## Construction and content

### Database content

Table 1 summarizes the content of DBSecSys. Using the available literature on Burkholderia species, we manually compiled a list of *B. mallei* proteins and their associated secretion systems. The resulting list contains five secretion system types associated with 143 *B. mallei* proteins, where each protein is associated with only one secretion system type. Additionally, as represented in DBSecSys, the list contains the available information about gene clusters associated with secretion systems, protein descriptions, and secretion system protein characterizations, e.g., effector or secretion apparatus proteins. *B. mallei* secretion system proteins are also characterized based on their association with one of 10 inferred pathogenic mechanisms of action.

**Table 1:** **Summary of the current content of DBSecSys**

Number of *B. mallei*	
proteins	143
virulence factors	21
virulence attenuation experiments	42
associated secretion systems	5
inferred mechanisms of action	10
**Number of host**	
species	2
proteins (human)	797
proteins (murine)	838
**Number of protein-protein interactions**	
human-*B. mallei* (experimental)	569
murine-*B. mallei* (experimental)	788
human-*B. mallei* (computational)	608
murine-*B. mallei* (computational)	435
human-human (experimental)	491
murine-murine (experimental)	36

In addition to pathogen proteins, DBSecSys contains information on 1,635 host (797 human and 838 murine) proteins, as well as information on 2,400 host-pathogen interactions between *B. mallei* and host proteins. Of these, 1,357 interactions have been detected experimentally: 569 human-*B. mallei* and 788 murine-*B. mallei* interactions. The remaining interactions (608 human-*B. mallei* and 435 murine-*B. mallei* interactions), have been determined computationally based on orthology between human and murine proteins provided in the National Center for Biotechnology Information (NCBI) HomoloGene database [17, 18]. In addition, DBSecSys contains a set of 491 protein-protein interactions (PPIs) [19] among 357 human proteins that interact with *B. mallei*, and 36 PPIs [20, 21] among 47 murine proteins that interact with *B. mallei*.

DBSecSys also provides information about 42 virulence attenuation (by genetic ablation) experiments for 27 of the 143 listed *B. mallei* proteins (21 of which are virulence factors). This information includes seven infection delivery routes, eight animal models used, and four observed virulence attenuation levels (full, moderate, mild, or no attenuation).

DBSecSys uses a standard nomenclature to describe secretion systems (see Table 2) and commonly used literature terms to define pathogenic mechanisms of action (see Table 3). All data in the database can be tracked to their experimental or computational source.

**Table 2:** **Description of secretion systems**

Secretion system type	Description
1*	- Consists of three protein subunits: the ATP-binding cassette (ABC) transporters, membrane fusion proteins, and outer membrane proteins.
	- Transports various proteins, e.g., RTX toxins and the lipases, as well as non-proteinaceous substrates, e.g., cyclic β-glucans and polysaccharides.
2*	- Represents a Sec/Tat-dependent system, as proteins that pass through this system must first reach the periplasm via either the general secretion route (Sec pathway) or the twin-arginine translocation pathway (Tat-pathway).
	- Sometimes used by Gram-negative bacteria type IV pili for their biogenesis.
3*	- Consists of machinery proteins (called injectisomes) and proteins that are secreted into a host cell (called effectors).
	- Sometimes consists of two or more gene clusters (pathogenicity islands).
	- Found in Gram-negative bacteria that interact with both plant and animal hosts.
4	- Can be divided into three types: *1*) a type IVA secretion system resembling the archetypal VirB/VirD4 system and consisting of conjugative plasmids F and RP4 (IncF and IncP); *2*) a type IVB secretion system also known as the intracellular multiplication/defect in organelle trafficking genes (icm/dot) system, consisting of conjugative plasmid R64; and *3*) a GI type that is, so far, associated exclusively with genomic islands.
	- Evolutionarily related to bacterial conjugation systems and capable of transporting both proteins and nucleic acids into host cells, as well as into other bacteria.
5*	- Also known as the autotransporter system.
	- Can be divided into three types: *1*) the archetypal bacterial proteins exported into the periplasm via the Sec system; *2*) trimeric proteins with a single beta barrel domain; and *3*) pairs of proteins in which one partner carries the beta barrel domain and the other partner is the secreted protein.
6*	- Consists of machinery proteins (called injectisomes) and proteins that are secreted into a host cell (called effectors).
	- Sometimes consists of two or more gene clusters (pathogenicity islands).
	- Nearly universally secretes two proteins: Hcp and VgrG.
7	- Used for the transport of extracellular proteins across the Gram-positive bacteria cell wall.
	- Often encoded in two or more gene clusters (pathogenicity islands).

*Associated with *B. mallei* (strain ATCC 23344) and included in DBSecSys.

**Table 3:** **Description of pathogenic mechanisms of action included in DBSecSys**

Name	Pathogens use this mechanism to:
Actin cytoskeleton rearrangement	Subvert the host cell cytoskeleton to promote attachment to host cell surface, internalization in the host cell, and to prevent uptake by phagocytic cells.
Actin-based motility	Bind to host actin, triggering actin polymerization on the pathogens’ surface and producing a mechanical force that propels them through the host cell and facilitates cell-to-cell spread.
Adhesion	Attach to host cell surface, promoting bacterial internalization in the host cell.
Apoptosis	Exert control on the processes that regulate apoptosis in the host.
Interference with signaling	Interfere with host signaling cascade, promoting their internalization in the host cell and intracellular survival.
Interference with the immune response	Down-regulate host inflammatory responses, promoting their internalization in the host cell and intracellular survival.
Invasion	Promote their ability to invade the host cell.
Multi-nucleated giant cell formation	Induce host cell fusion and multi-nucleated giant cell formation.
Phagosomal escape and evasion of autophagy	Ensure bacterial escape from endocytic vesicles, as well as to evade autophagosome, ensuring the pathogens’ intracellular survival and cell-to-cell spread.
Ubiquitination - deubiquitination	Interfere with host ubiquitination processes to attenuate host immune response, to prevent their degradation, and to ensure their destruction when no longer required for establishing the infection.

### Data sources

The list of *B. mallei* proteins associated with each secretion system was compiled using the results of *B. mallei* experimental studies [22–24], computational predictions based on orthology, and *de-novo* computational predictions. We used orthology to infer *B. mallei* proteins associated with secretion systems based on the results of *B. pseudomallei* experimental studies, as these two species share a large number of genes with high sequence similarity [22–27]. We used only a subset of predicted *B. mallei* secretion system proteins that has been shown to interact with host proteins experimentally [28].

Twenty-four *B. mallei* proteins associated with secretion systems participate in PPIs with human and murine hosts [28]. Given that the PPI detection experiments did not exhaustively screen for all possible host-*B. mallei* interactions, we used human-murine orthology information [17, 18] to infer additional interactions. This procedure generated a list of experimentally and computationally identified human- and murine-*B. mallei* PPIs associated with secretion systems. Furthermore, to account for the relationship among host proteins that interact with *B. mallei* proteins, we used information from host PPI networks. For human PPIs, we used data from an experimentally detected PPI network compiled by Yu *et al.* [19], while for murine PPIs, we used data from an experimentally detected PPI network available in the BIOGRID database (Release 3.2.105) [20, 21].

We compiled the list of *B. mallei* pathogenic mechanisms of action based on the results of *B. mallei* experimental studies [22, 29] and the computational predictions using orthology and host-pathogen PPI data. We used information about orthology between *B. mallei* and *B. pseudomallei* proteins to infer mechanisms of action experimentally identified for *B. pseudomallei* [30–32]. We inferred *B. mallei* mechanisms of action from host-pathogen PPIs as follows. First, we surveyed the available pathogenicity literature for associations between specific host protein functions and pathogenic mechanisms of action. Then, for each pathogenic mechanism of action, we compiled a set of its corresponding host proteins' functions. Next, for each *B. mallei* protein present in host-*B. mallei* PPI data [28], we counted the number of its host-interacting partners that were annotated with at least one function associated with each of the mechanisms. If a *B. mallei* protein interacted with *≥ 3* host proteins that corresponded to a single mechanism, we assigned the protein to that mechanism of action.

Finally, using literature information, we manually compiled a list of *B. mallei* secretion system proteins that have been experimentally evaluated for virulence attenuation (by genetic ablation) in an animal model. The list contains proteins identified in *B. mallei* studies [28] and proteins computationally inferred from *B. pseudomallei* studies using orthology information [30, 33, 34].

We used NCBI [17, 18] and Uniprot [35] to annotate pathogen and host proteins, e.g., protein names and sequence information. For functional annotation of host proteins, we used Gene Ontology (GO) terms [36] and Kyoto Encyclopedia of Genes and Genomes (KEGG) pathways [37, 38].

### Software architecture

We developed the DBSecSys database and Web interface using a three-tier architecture comprising a backend database, controller, and presentation tiers. The first tier consists of an Oracle database server that stores the data contained in the DBSecSys database using a relational schema. We developed the controller and presentation tiers using Java Platform, Enterprise Edition 7, JavaServer Faces 2, and ICEfaces 3 technologies. The presentation tier comprising a Web interface also uses JBrowse [39–41], D3.js [42], NVD3.js [43], and Cytoscape.js [44] JavaScript libraries to create detailed interactive visualizations of genes on the reference sequence and of protein-protein interactions. All visualizations are based on modern Web standards and do not require any plugins. The DBSecSys Web application is hosted on an Apache Tomcat Web server at: https://applications.bhsai.org/dbsecsys.

## Utility and discussion

**Figure 1:**
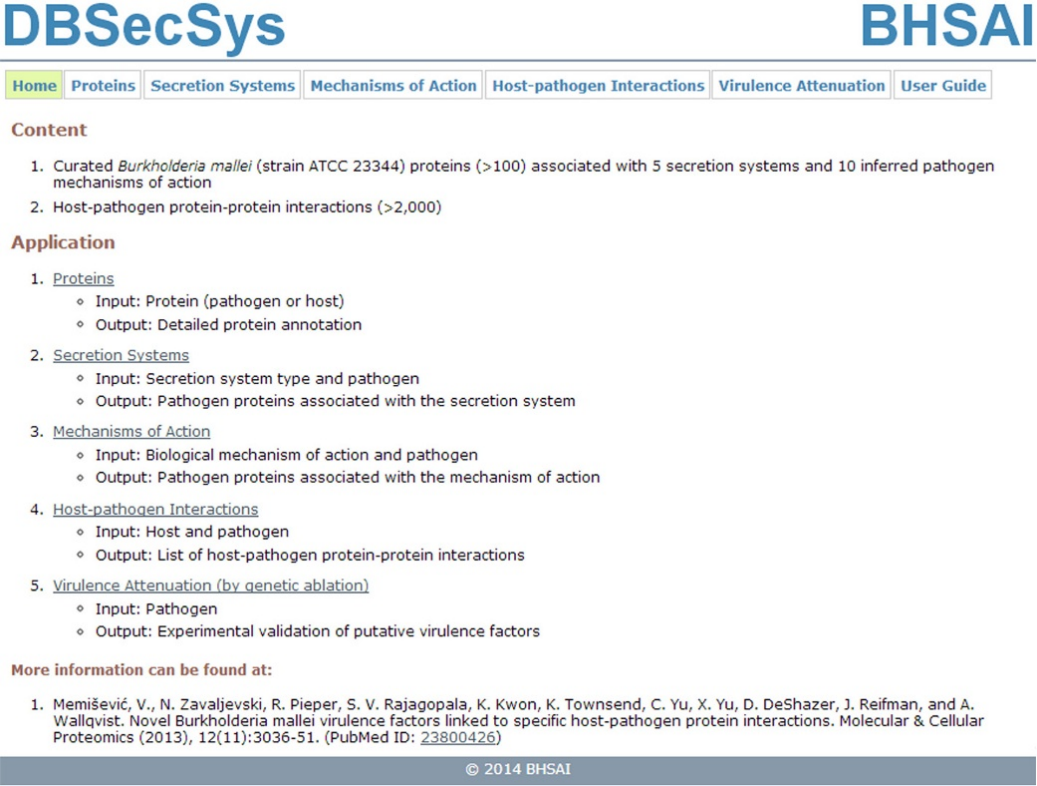
**DBSecSys home page.** The Web interface of DBSecSys provides features for browsing, querying, visualizing, and downloading detailed information about *Burkholderia mallei* (strain ATCC 23344) proteins associated with secretion systems, their involvement in virulence, their host protein targets, and their mechanisms of action inferred from host-pathogen interaction data and literature review.

**Figure 2:**
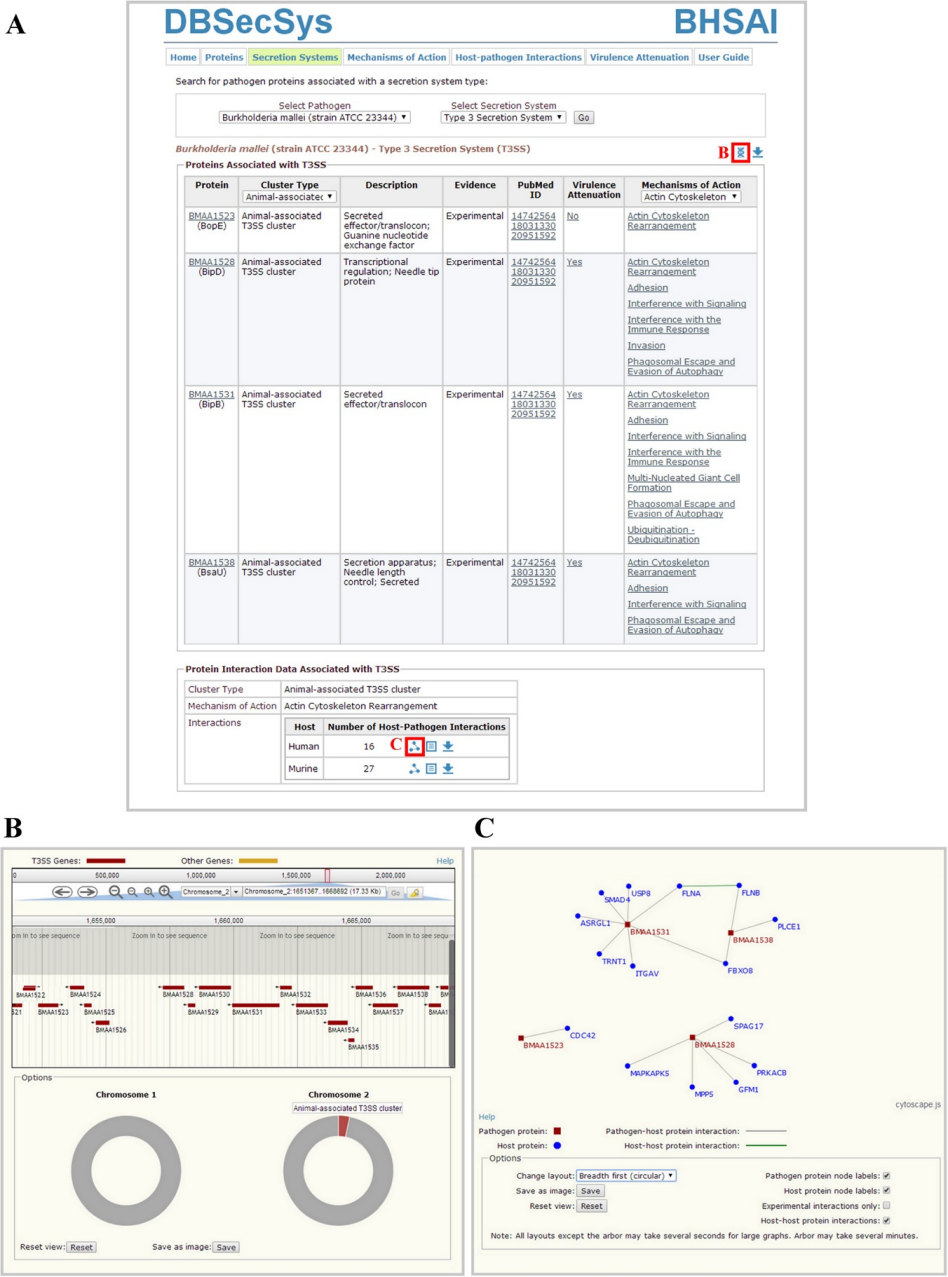
**Secretion systems search results. (A)** A search for *B. mallei* (strain ATCC 23344) type 3 secretion system proteins yields a list of pathogen proteins associated with that secretion system. The content of the list can be filtered by cluster type and mechanisms of action, e.g., by the animal-associated T3SS cluster and the actin cytoskeleton rearrangement mechanism. **(B)** The genome browser shows the location of the bacterial proteins associated with the secretion system on the bacterial reference sequence. **(C)** Users can visualize host-pathogen protein-protein interactions (PPIs) with the help of the PPI browser. Interactions are represented in the form of networks, where proteins correspond to network nodes (blue circles and red squares), and interactions between those proteins correspond to network edges (gray and green lines).

At its core, DBSecSys contains information about *B. mallei* secretion system proteins, their inferred pathogenic mechanisms of action, host protein interacting partners, and virulence attenuation experiments. A Web-based graphical user interface presents the DBSecSys data with interactive search and visualization capabilities. It provides the ability to browse, query, and visualize these data in multiple ways, allowing users to explore secretion systems through pathogen proteins, as well as their host-interacting partners (Figures 1 and 2). All data within DBSecSys are cross-linked. This feature facilitates efficient navigation between different DBSecSys pages. It also allows users to apply retrieved results as queries for new searches, minimizing the need to navigate to a designated query page. DBSecSys query results can be filtered and downloaded in a tab-delimited format. Host-pathogen PPIs are available for download in the Proteomics Standards Initiative Molecular Interactions (PSI-MI) Tab 2.5 format [45]. DBSecSys also provides links to external resources for detailed information about individual proteins (NCBI [17, 18], Uniprot [35], GO [36], and KEGG [37, 38]). For all experimental data and observations contained in the database, DBSecSys provides links to their original sources and publications through PubMed.

DBSecSys queries are anchored around five applications (Figure 1), which search for *1*) pathogen protein or host protein annotation, *2*) all pathogen proteins associated with a secretion system, *3*) all pathogen proteins associated with the pathogen’s mechanism of action, *4*) host-pathogen interactions, and *5*) experimentally screened virulence factors.

### Application 1: Study of individual proteins

Users can study individual (pathogen or host) proteins on the “Proteins” page of the Web interface. For example, users can search either for a *B. mallei* protein to determine its association with a secretion system, or for a host (human or murine) protein to determine whether it interacts with a pathogen protein associated with a secretion system. Protein searches can be performed using one of the following known protein/gene identifiers: Locus Tag, Name, Uniprot ID, Uniprot Name, Gene ID, GenInfo Identifier (GI), and KEGG ID.

When the query protein is represented in the database, DBSecSys returns its detailed annotation information, including protein/gene identifiers from different annotation databases (see above), chromosomal location, amino acid sequence information, and corresponding GO [36] and KEGG [37, 38] annotations. All annotation information is linked with its external source. If the host-*B. mallei* interactions data is available for the queried protein, DBSecSys provides a list of host-pathogen PPIs for that protein. Additionally, if the queried protein is a pathogen protein, the database provides information about its associated secretion system, inferred mechanisms of action, and, if it was tested for virulence attenuation, information about its effect on virulence. Conversely, if the queried protein is a host protein, DBSecSys provides information about pathogen proteins that target this host protein, e.g., the secretion systems with which they are associated and their inferred mechanisms of action.

### Application 2: Study of pathogen proteins associated with a secretion system

Users can study pathogen proteins associated with a specific secretion system on the “Secretion Systems” page of the Web interface. This page allows users to select one of the secretion systems associated with *B. mallei* and list all pathogen proteins associated with it. DBSecSys retrieves a list of *B. mallei* proteins associated with the queried secretion system and the information about their role, the pathogenicity island/cluster type (for the secretion systems that are encoded in two or more gene clusters), details about their associated mechanism(s) of action, and details about the corresponding virulence attenuation experiments. If host-*B. mallei* interaction data are available for the listed *B. mallei* proteins, DBSecSys also retrieves the corresponding host-pathogen PPIs. Users have the option to filter the retrieved list of proteins based on a secretion system cluster or a mechanism of action. DBSecSys also filters the corresponding list of host-pathogen PPIs, ensuring that it contains only proteins that satisfy user-specified criteria (filters).Figure 2A shows a list of proteins associated with the type 3 secretion system that are filtered based on the cluster type (animal-associated cluster) and the mechanism of action association (actin cytoskeleton rearrangement), while Figure 2B and C show visualization options for proteins associated with secretion system clusters and their associated host-pathogen PPIs, respectively.

### Application 3: Study of pathogen proteins associated with an inferred pathogenic mechanism of action

Users can also study pathogen proteins associated with a specific pathogenic mechanism of action using the “Mechanisms of Action” page of the Web interface. The querying process for this application is similar to the one for Application 2, and the result of a query is a list of *B. mallei* proteins, their associated secretion system types, and detailed information about their association with the queried mechanism of action. The retrieved list can be filtered by secretion system type. If host-*B. mallei* interaction data are available for the listed *B. mallei* proteins, DBSecSys also retrieves their corresponding host-pathogen PPIs.

### Application 4: Host-pathogen interactions associated with secretion systems

DBSecSys allows users to browse all available host-pathogen interactions in the database using the “Host-Pathogen Interactions” page. This page gives users an option to retrieve host-specific interactions or all host-*B. mallei* interactions. Additionally, this page allows users to select the type of interactions they want to retrieve – only experimentally detected PPIs (the default option) or experimentally detected and computationally predicted PPIs. The resulting list of host-*B. mallei* interactions can be filtered based on host and pathogen protein names. Subsets of host-*B. mallei* PPIs can also be accessed through multiple other pages on the DBSecSys Web interface, e.g., through searches by protein, secretion system, or mechanisms of action.

### Application 5: Experimentally screened virulence factors associated with secretion systems

DBSecSys contains detailed information about *B. mallei* secretion system proteins tested for virulence attenuation (by genetic ablation) in animal model experiments, which can be queried using the “Virulence Attenuation” page on the Web interface. The resulting list of proteins contains information about the virulence attenuation level, animal model and infection route used, and the associated secretion system type. Users can filter the list of proteins based on the secretion system type, animal model, and infection route.

### Data visualization

The DBSecSys Web interface provides two visualization tools: *1*) an interactive genome browser for displaying pathogen genes on the reference sequence and *2*) an interactive network visualization tool for displaying host-pathogen PPIs.

Figure 2B shows the genome browser that displays the *B. mallei* reference sequence and highlights the chromosomal location of secretion system genes and gene clusters, allowing users to locate the positions of genes associated with the secretion systems and interactively explore different parts of the sequence. It also enables users to study the individual secretion system genes in the context of the adjacent genes that are not associated with secretion systems, and to study multiple genes that are part of a specific secretion system or a secretion system cluster, or are associated with the same mechanism of action.Figure 2C shows the PPI browser that provides visualization of host-pathogen PPIs in the form of networks, where proteins are represented as network nodes and interactions between proteins are represented as network edges. Visualization of host-pathogen PPIs may help users identify connectivity patterns underlying these interactions. By default, the PPI browser displays the interactions using a circular breadth-first search layout. However, users can choose one of the provided layout options (breadth first linear, circle, and force-directed arbor layouts) or interactively move and rearrange all nodes and edges. The PPI browser also allows users to select additional visualization options, e.g., to display or hide computationally predicted PPIs and to visualize the interactions between host proteins by displaying host PPIs.

Both tools provide zoom in/out options and the ability to save the resulting view as an image. Additionally, visualization tools provide contextual pop-up displays that allow users to efficiently retrieve more information about proteins directly from the visualization tools, e.g., information provided by NCBI (genome browser) or information about the protein secretion system type and virulence attenuation (PPI browser).

### Database updates

The database content will be updated periodically to include new experimental and computational evidence. Additionally, the database will be expanded to include additional pathogens, their corresponding secretion systems, mechanisms of action, and virulence attenuation information. Before each update, the current state of the database will be frozen and archived.

## Conclusions

We developed a curated database of bacterial secretion system proteins and their host factors. Currently, the database contains comprehensive information about *B. mallei* proteins associated with secretion systems, their involvement in virulence, their host targets (proteins), and their mechanisms of action inferred from literature and host-pathogen interaction data. Systematical organization of experimental and computational data allows for efficient data retrieval, browsing, querying, and visualization, and enables users to study secretion systems not only through characterization of the corresponding pathogen proteins but also through characterization of host-interacting partners. Together, these features make DBSecSys a unique resource for *B. mallei* secretion system research.

## Availability and requirements

The Web-enabled DBSecSys database is freely accessible at https://applications.bhsai.org/dbsecsys. The DBSecSys Web interface has been tested in the following Web browsers: Google Chrome (version 31), Microsoft Internet Explorer (version 10), and Mozilla Firefox (version 25). The “User Guide” page of the DBSecSys Web interface includes a step-by-step description of all DBSecSys features.

## Abbreviations

DBSecSys: Database of *Burkholderia mallei* Secretion Systems
GO: Gene Ontology
KEGG: Kyoto Encyclopedia of Genes and Genomes
NCBI: National Center for Biotechnology Information
PPI: Protein-protein interaction
PSI MI: Proteomics Standards Initiative Molecular Interactions.
